# Efficient Spike-Driven Learning With Dendritic Event-Based Processing

**DOI:** 10.3389/fnins.2021.601109

**Published:** 2021-02-19

**Authors:** Shuangming Yang, Tian Gao, Jiang Wang, Bin Deng, Benjamin Lansdell, Bernabe Linares-Barranco

**Affiliations:** ^1^School of Electrical and Information Engineering, Tianjin University, Tianjin, China; ^2^Department of Bioengineering, University of Pennsylvania, Philadelphia, PA, United States; ^3^Microelectronics Institute of Seville, Seville, Spain

**Keywords:** spiking neural network, credit assignment, dendritic learning, neuromorphic, spike-driven learning

## Abstract

A critical challenge in neuromorphic computing is to present computationally efficient algorithms of learning. When implementing gradient-based learning, error information must be routed through the network, such that each neuron knows its contribution to output, and thus how to adjust its weight. This is known as the credit assignment problem. Exactly implementing a solution like backpropagation involves weight sharing, which requires additional bandwidth and computations in a neuromorphic system. Instead, models of learning from neuroscience can provide inspiration for how to communicate error information efficiently, without weight sharing. Here we present a novel dendritic event-based processing (DEP) algorithm, using a two-compartment leaky integrate-and-fire neuron with partially segregated dendrites that effectively solves the credit assignment problem. In order to optimize the proposed algorithm, a dynamic fixed-point representation method and piecewise linear approximation approach are presented, while the synaptic events are binarized during learning. The presented optimization makes the proposed DEP algorithm very suitable for implementation in digital or mixed-signal neuromorphic hardware. The experimental results show that spiking representations can rapidly learn, achieving high performance by using the proposed DEP algorithm. We find the learning capability is affected by the degree of dendritic segregation, and the form of synaptic feedback connections. This study provides a bridge between the biological learning and neuromorphic learning, and is meaningful for the real-time applications in the field of artificial intelligence.

## Introduction

Learning requires assigning credit to each neuron for its contribution to the final output (Bengio et al., 2015; Lillicrap et al., 2016). How a neuron determines its contribution is known as the credit assignment problem. In particular, the training of deep neural networks is based on error back-propagation, which uses a feedback pathway to transmit information to calculate error signals in the hidden layers. However, neurophysiological studies demonstrate that the conventional error back-propagation algorithm is not biologically plausible. One problem is known as weight transport: backpropagation utilizes a feedback structure with the exact same weights as the feed-forward pathway to communicate gradients (Liao et al., 2016). This symmetric feedback structure has not been proven to exist in biological neural circuit. Several studies have presented solutions to modify or approximate the backpropagation algorithm in a more biologically plausible manner (Lee et al., 2015; Scellier and Bengio, 2017; Lansdell and Kording, 2019; Lansdell et al., 2019). In fact, active channels in dendrites can drive different forms of spiking activities (Schmolesky et al., 2002; Larkum, 2013). A potential solution is thus to segregate signals into dendritic compartments, so that the credit signals can be kept separate from other ongoing computation (Richards and Lillicrap, 2019). Recent work shows how spiking neural networks can implement feedback structures that allow efficient solving of the credit assignment problem by dendritic computation (Urbanczik and Senn, 2014; Wilmes et al., 2016; Bono and Clopath, 2017; Guerguiev et al., 2017). Further, other work has shown that even feedback systems that crudely approximate the true feedback weights can solve some learning tasks (Zenke and Ganguli, 2018; Lee et al., 2020). Together these works show that the credit assignment problem can be largely solved by biologically plausible neural systems.

An ongoing challenge in neuromorphic computing is to present general and computationally efficient algorithms of deep learning. Previous works have shown how neuromorphic approaches for deep learning can be more efficient compared to Von Neumann architecture (Esser et al., 2015; Indiveri et al., 2015; Neftci et al., 2017). However, these systems have yet to be fully realized. By design, learning in neuromorphic hardware operates under similar constraints to learning in biological neural networks. The credit assignment problem, and the problem of weight transport also manifest in this setting: neuromorphic learning systems that do not require weight transport enjoy less data transfer between components. In this way, biologically plausible approaches to deep learning can also be used to make neuromorphic computing more efficient. Previous neuromorphic systems have been presented for high-performance brain-inspired computation, providing tests of biological learning models and real-time applications (Qiao et al., 2015; Yang et al., 2015, 2018, 2020, 2021).

Recent proposals for solutions to the credit assignment problem have not been considered in neuromorphic computing. Here we present a novel dendritic event-based processing (DEP) algorithm to facilitate the efficient implementation of the credit-assignment task on neuromorphic hardware. The presented DEP algorithm is inspired by the primary sensory areas of the neocortex, providing the segregation of feed-forward and feedback information required to compute local error signals and to solve the credit assignment problem. In the DEP algorithm, a binarization method and a dynamic fixed-point solution are presented for the efficient implementation of deep learning. The paper is organized as follows: section “Introduction” describes the preliminaries of this study, including neuromorphic computing and the spiking neural network (SNN) model. Learning with stochastic gradient descent (SGD) in spiking neural networks is introduced and explained in section “Materials and Methods.” Section “Results” presents the experimental results. And finally, the discussions and conclusions are proposed in sections “Discussion” and “Conclusion,” respectively.

## Materials and Methods

### Learning With Dendrites in Event-Driven Manner

Learning needs neurons to receive signals to assign the credit for behavior. Since the behavioral impact in early network layers is based on downstream synaptic connections, credit assignment in multi-layer networks is challenging. Previous solutions in artificial intelligence use the backpropagation of error algorithm, but this is unrealistic in the neural systems. Rather than requiring weight transport, current biologically plausible solutions to the credit assignment problem use segregated feed-forward and feedback signals (Lee et al., 2015; Lillicrap et al., 2016). In fact, the cortico-cortical feedback signals to pyramidal neurons can transmit the necessary error information. These works show how the circuitry needed to integrate error information may exist within each neuron. The idea is that both feed-forward sensory information in the neocortex and the higher-order cortico-cortical feedback are received by different dendritic compartments, including basal and apical dendrites (Spratling, 2002). In a pyramidal cell, distal apical dendrites are distant from the soma, and communicate with the soma based on active propagation using the apical dendritic shaft, driven predominantly by voltage-gated calcium channels (Katharina et al., 2016). Further, there exist dynamics of plateau potentials that generate prolonged upswings in the membrane potential. These are based on the nonlinear dynamics of voltage-gated calcium current, and drive bursting at the soma (Larkum et al., 1999). The plateau potentials of the apical dendritic activities can induce learning in pyramidal neurons *in vivo* (Bittner, 2015).

**FIGURE 1:**
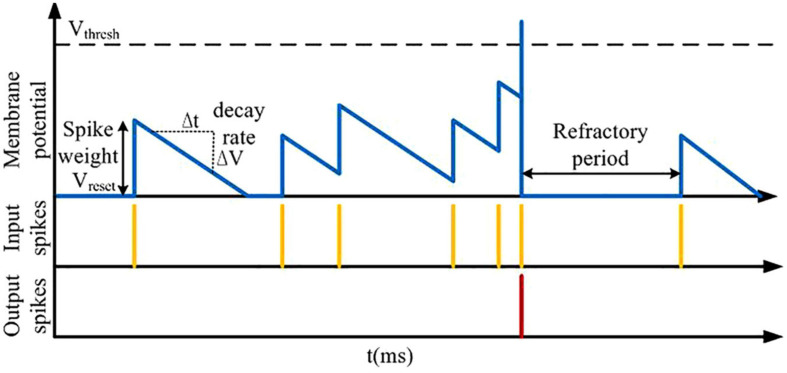
Event-driven neural computing, showing the process of synaptic weights and time affecting the neural membrane potential and the refractory period.

Inspired by these phenomena, a previous study has proposed a learning algorithm with segregated dendrites (Guerguiev et al., 2017). Based on this work, an efficient learning algorithm for neuromorphic learning is presented in this study. The idea is that the basal dendritic compartment is coupled to the soma for processing bottom-up sensory information, and the apical dendritic compartment is used to process top-down feedback information to calculate credit assignment and induce learning using plateau potentials. The basic computing unit we use on the large-scale conductance-based spiking neural network (LaCSNN) system is based on the integrate-and-fire (IF) principle. As shown in Figure 1, the simple spiking behaviors of the IF neurons can be triggered by excitatory input spikes. The new state of the neural membrane potential with an input arriving is determined by the last updating time and the previous state. Thus, the event-driven neuron only updates when an input spike is received. Then the membrane potential decay after the last update is retroactively calculated and applied. The synaptic weight is then used to contribute to the resulting membrane potential. A spike event is emitted when the membrane potential exceeds a spike threshold, and then the neural activity is reset and mutual inhibition with coupled neurons is realized. Finally the membrane potential and spiking event are written to memory to store the network state of the next update of neural activity.

### Network Architecture With SGD Algorithm

**FIGURE 2:**
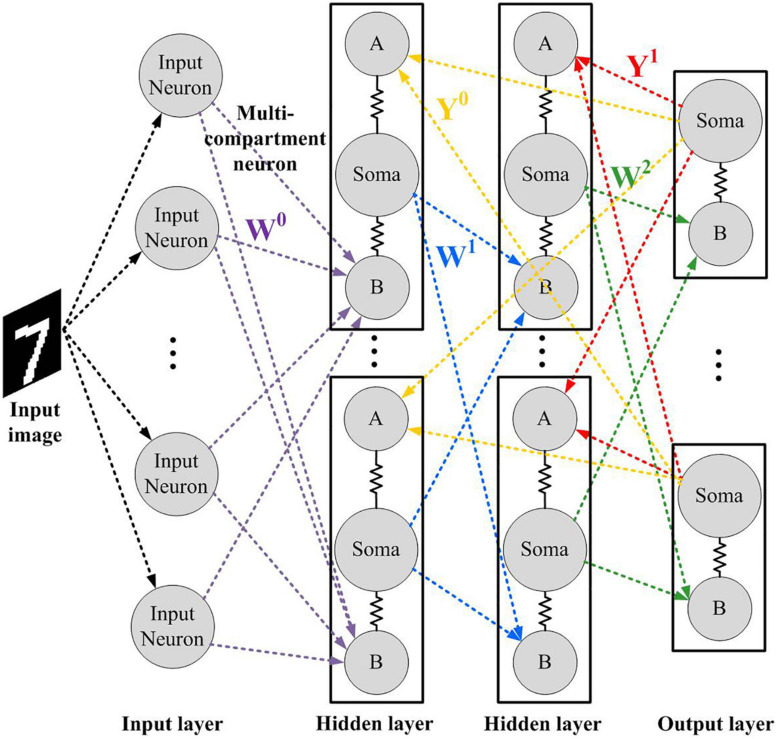
The network architecture in this study. Matrices *Y* and *W* represent the feedback and feed-forward synaptic weight matrix, respectively.

The network diagram utilizes the SNN model in the previous study by Guerguiev et al. (2017) as shown in Figure 2, which consists of an input layer with *m* = 784 neurons, a hidden layer with *n* = 500 neurons, and an output layer with *p* = 10 neurons. Since our primary interests are in the realization of neuromorphic networks, the proposed model is restricted to discrete systems based on the Euler method, where *N* is the time step for discretization. This way of representation is popular in the hardware implementation of spiking neural networks because of its feasibility of implementation and routing. Poisson spiking neurons are used in the input layer, whose firing rate is determined by the intensity of image pixels ranging from 0 to *Φ_*max*_*. In the hidden layer, neurons are modeled using three functional compartments, which are basal dendrites, apical dendrites and soma. The membrane potential of the *i*th neuron in the hidden layer is updated as follows:

(1) $$\begin{array}{c} \tau \,\frac{V_{i}^{0}\,\left(N+1\right)-V_{i}^{0}\,\left(N\right)}{\Delta \,T}=-V_{i}^{0}\,\left(N\right)+\frac{g_{b}}{g_{l}}\,\left(V_{i}^{0\,b}\,\left(N\right)-V_{i}^{0}\,\left(N\right)\right)\\ +\frac{g_{a}}{g_{l}}\,\left(V_{i}^{0\,a}\,\left(N\right)-V_{i}^{0}\,\left(N\right)\right) \end{array}$$

where *g*_*l*_, *g*_*b*_, and *g*_*a*_ stand for the leak conductance, the basal dendrites conductance, and the apical dendrite conductance, and Δ*T* is the integration step. The superscript “0,” “a,” and “b” represent hidden layer, basal dendrite and apical dendrite. The parameter *τ* = *C*_*m*_/*g*_*l*_, is a time constant, where *C*_*m*_ represents the membrane capacitance. The variables *V*^0^, *V*^0^*^*a*^*, and *V*^0^*^*b*^* represent the membrane potentials of soma, apical dendrite and basal dendrite, respectively. The dendritic compartments are defined as weighted sums for the *i*th hidden layer neuron as follows:

(2) $$\left\{ \begin{array}{c} V_{i}^{0\,b}\,\left(N\right)=\sum_{j=1}^{m}W_{i\,j}^{0}\,s_{j}^{i\,n\,p\,u\,t}\,\left(N\right)+b_{i}^{0}\\ V_{i}^{0\,a}\,\left(N\right)=\sum_{j=1}^{p}Y_{i\,j}\,s_{j}^{1}\,\left(N\right) \end{array}\right.$$

where *W*_*ij*_^0^ and *Y*_*ij*_ are synaptic weights in the input layer and feedback synapses, respectively. The constant *b*_*i*_^0^ is defined as a bias term, and *s*^*input*^ and *s*^1^ are the filtered spiking activities in the input layer and output layer, respectively. The variable *s*^*input*^ is calculated based on the following equations as

(3) $$s_{j}^{i\,n\,p\,u\,t}\,\left(t\right)=\sum_{k}\kappa \,\left(t-t_{j\,k}^{i\,n\,p\,u\,t}\right)$$

where *t_*jk*_^*input*^* represents the *k*th spiking time of the input neuron *j*, and the response kernel is calculated as

(4) $$\kappa \,\left(t\right)=\left(e^{-t\,/\,\tau_{L}}-e^{-t\,/\,\tau_{s}}\right)\,\Theta \,\left(t\right)\,/\,\left(\tau_{L}-\tau_{s}\right)$$

where *τ*_*L*_ and *τ*_*s*_ are long and short time constants, and Θ is the Heaviside step function. The filtered spike trains at apical synapses *s*^1^ is modeled based on the same method. The spiking activities of somatic compartments are based on Poisson processes, whose firing rates are based on a non-linear sigmoid function *σ*(.) for the *i*th hidden layer neuron as follows:

(5) $$\Phi_{i}^{0}\,\left(N\right)=\phi_{\max}\,\sigma \,\left(V_{i}^{0}\,\left(N\right)\right)=\phi_{\max}\,\frac{1}{1+e^{-V_{i}^{0}\,\left(N\right)}}$$

where *Φ_*max*_* represents the maximum firing rates of neurons.

### Plateau Potentials and Weight Updates

**FIGURE 3:**
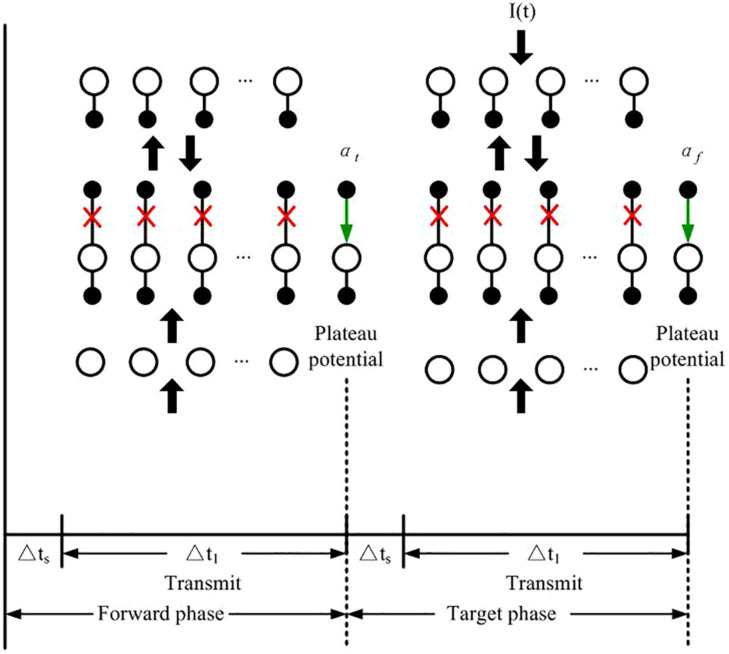
Network computing phases for learning proposed by Guerguiev et al. The green arrows represent the signal transmission from apical dendrite to soma, and red crosses stand for the disconnection between apical dendrite and somatic compartment. The black arrows represent the transmission of spike signals between layers.

Based on the learning algorithm of Guerguiev et al., two phases are alternated to train the network: the forward and target phases as shown in Figure 3. In the forward phase *I*_*i*_(*t*) = 0, while it induces any given neuron *i* to spike at maximum firing rate or be silent according to the category of the current input image when the network undergoes target phase. At the end of the forward phase and the target phase, the set of plateau potentials *α_*t*_* and *α_*f*_* are calculated, respectively.

At the end of each phase, plateau potentials are calculated for apical dendrites of hidden layer neurons, which are defined as follows

(6) $$\left\{ \begin{array}{c} \tau \,\frac{V_{i}^{1}\,\left(N+1\right)-V_{i}^{1}\,\left(N\right)}{\Delta \,T}=-V_{i}^{1}\,\left(N\right)+I_{i}\,\left(N\right)\\ +\frac{g_{d}}{g_{l}}\,\left(V_{i}^{1\,b}\,\left(N\right)-V_{i}^{1}\,\left(N\right)\right)\,\\ V_{i}^{1\,b}\,\left(N\right)=\sum_{i=1}^{m}W_{i\,j}^{1}\,s_{j}^{0}\,\left(N\right)+b_{i}^{1} \end{array}\right.$$

where *t*_1_ and *t*_2_ represent the end times of the forward and target phases, respectively. Δ*t*_*s*_ = 30 ms represents the settling time for the membrane potentials, and Δ*t*_1_ and Δ*t*_2_ are formulated as follows

(7) $$\left\{ \begin{array}{c} \Delta \,t_{1}=t_{1}-\left(t_{0}+\Delta \,t_{s}\right)\\ \Delta \,t_{2}=t_{2}-\left(t_{1}+\Delta \,t_{s}\right) \end{array}\right..$$

The temporal intervals between plateaus are sampled based on an inverse Gaussian distribution randomly. Although the system computes in phases, the specific length of the phases is not vital, provided there has been a long enough time to integrate the input currents.

### Learning With Feedback Driven Plateau Potentials

During the forward phase, an image is presented to the input layer without teaching current at the output layer between time *t*_0_ to *t*_1_. At *t*_1_ a plateau potential is computed in the hidden layer neurons and the target phase begins. During the target phase the image is also presented into the input layer that also receives teaching current, forcing the correct neuron in the output layer to its maximum firing rate while others are silent. At time *t*_2_ another set of plateau potentials in the hidden layers are computed. Plateau potentials for the end of both the forward and the target phases are calculated as follows

(8) $$\left\{ \begin{array}{c} \alpha_{i}^{f}=\sigma \,\left(\frac{1}{\Delta \,t_{1}}\,\int_{t_{1}-\Delta \,t_{1}}^{t_{1}}V_{i}^{0\,a}\,\left(N\right)\,dt\right)\\ \alpha_{i}^{t}=\sigma \,\left(\frac{1}{\Delta \,t_{2}}\,\int_{t_{2}-\Delta \,t_{2}}^{t_{2}}V_{i}^{0\,a}\,\left(N\right)\,dt\right) \end{array}\right.$$

where Δ*t*_*s*_ represents a time delay of the network dynamics before integrating the plateau, and Δ*t_*i*_* = *t*_*i*_ − (*t_*i*__–__1_* + Δ*t*_*s*_). The superscript “*t*” and “*f*” represent target and forward phases, respectively.

The basal dendrites in the hidden layer update the synaptic weights *W*_0_ based on the minimization of the loss function as follows

(9) $$L^{0}={||\phi^{0^{*}}-\phi_{\text{max}}\,\sigma \,\left({\bar{v}}^{0}\,f\right)||}_{2}^{2}.$$

And the target firing rate is defined as

(10) $$\phi_{i}^{0,*}={\bar{\Phi }}_{i}^{0^{f}}+\alpha_{i}^{t}-\alpha_{i}^{f}$$

where the variable and are plateau potentials in the forward and target phases. It should be noted that as long as neural units calculate averages after the network has reached a steady state, and the firing rates of the neurons are in the linear region of the sigmoid function, then we have the following equation for the hidden layer as:

(11) $$\phi_{\max}\,\sigma \,\left({\bar{V}}^{0\,f}\right)\approx \phi_{\max}\,{\bar{\sigma \,\left(V^{0}\right)}}^{f}={\bar{\phi^{0}}}^{f}$$

Then the formulation can be obtained as

(12) $$L^{0}\approx {||\alpha^{t}-\alpha^{f}||}_{2}^{2}.$$

And the formulation can be described as follows

(13) $$\left\{ \begin{array}{c} \frac{\partial L^{0}}{\partial W^{0}}\approx -k_{b}\,\left(\alpha^{t}-\alpha^{f}\right)\,\phi_{\max}\,\sigma^{\prime }\,{\left({\bar{V}}^{0\,f}\right)}^{\circ }\,{\bar{s}}^{i\,n\,p\,u\,t\,f}\\ \frac{\partial L^{0}}{\partial b^{0}}\approx -k_{b}\,\left(\alpha^{t}-\alpha^{f}\right)\,\phi_{\max}\,\sigma^{\prime }\,\left({\bar{V}}^{0\,f}\right) \end{array}\right.$$

where the constant *k*_*b*_ is given as

(14) $$k_{b}=g_{b}/\left(g_{l}+g_{b}+g_{a}\right).$$

In this study, *Φ*^0*^ is treated as a fixed state for the hidden layer neurons to learn. The synaptic weights of basal dendrites are updated to descend the approximation of the gradient as follows

(15) $$\left\{ \begin{array}{c} W^{0}\to W^{0}-\eta^{0}\,P^{0}\,\frac{\partial L^{0}}{\partial W^{0}}\\ b^{0}\to b^{0}-\eta^{0}\,P^{0}\,\frac{\partial L^{0}}{\partial b^{0}} \end{array}\right..$$

In the target phase the activity is also fixed and no derivatives are used for the membrane potentials and firing rates. The feedback weights are held fixed.

### Piecewise Linear Approximation (PWL) for Digital Neuromorphic Computing

**FIGURE 4:**
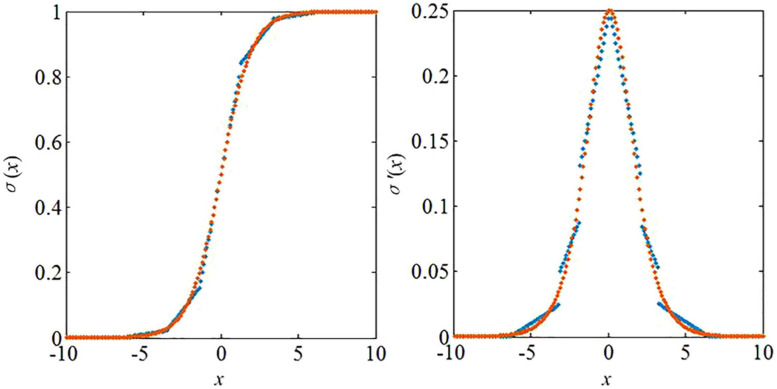
PWL functions in the proposed algorithms.

Here we simplify the above model for efficient use in neuromorphic architectures. In order to avoid the complicated computation induced by nonlinear functions, the PWL approach is used in this study. Both the functions*σ*(*x*) and *σ*’(*x*) are modified based on the PWL method, which can be formulated as follows

(16) $$f_{P\,W\,L}=\left\{ \begin{array}{c} a_{1}\,x+b_{1},\mathrm{when}\,x\leq s_{1}\\ a_{2}\,x+b_{2},\mathrm{when}\,s_{1}<x\leq s_{2}\\ \ldots \\ a_{i}\,x+b_{i},\mathrm{when}\,x>s_{i-1} \end{array}\right.$$

where *a*_*i*_ and *b*_*i*_ are the slope and intercept of the modified PWL function *f_*ow*__*l*_*, respectively (*i* = 1, 2,…, *n*). Since the range of the segment points are constrained, an exhaustive search algorithm is used in the determination of the PWL functions. The determination of the coefficient values are based on an error evaluation criterion as follows

(17) $$C\,F_{R\,E}=\frac{1}{n}\,\sqrt{\sum_{i=1}^{n}\frac{{\left(f_{o\,r\,i}\,\left(i\right)-f_{P\,W\,L}\,\left(i\right)\right)}^{2}}{f_{o\,r\,i}\,{\left(i\right)}^{2}}}$$

where *n* represents the total sampling points, and *f*_*ori*_ represents the original function. If the modified function cannot meet the accuracy requirement represented by *CF*_*RE*_, its segment number will be added by 1 until it can be guaranteed. Since the multiplication operation is replaced by “adder” and “shifter” in the proposed study, the coefficient value *a*_*i*_ in the PWL functions should be a power of 2 (for example: 1, 2, 4 or 0.5, 0.25, etc.). The parameter values of the PWL methods are listed in Table 1. The PWL functions are depicted in Figure 4.

**TABLE 1:** Parameter values of the PWL methods.

***σ*(*x*)**	***A***	***b***	**Condition**
*i* = 1	0.0078125	0.05	*x*≦−3.4
*i* = 2	0.0625	0.24	−3.4 < *x*≦−1.3
*i* = 3	0.25	0.5	−1.3 < *x*≦1.3
*i* = 4	0.0625	0.76	1.3 < *x*≦3.4
*i* = 5	0.0078125	0.95	3.4 < *x*
*i* = 6	0	0.9999	*σ*(*x*)≦0
*i* = 7	0	0.0001	*σ*(*x*)≧1
***σ*’(*x*)**	***A***	***b***	**Condition**
*i* = 1	0.0078125	0.05	*x*≦−3.2
*i* = 2	0.03125	0.15	−3.2 < *x*≦−2
*i* = 3	0.0625	0.25	−2 < *x*≦0
*i* = 4	−0.0625	0.25	0 < *x*≦2
*i* = 5	−0.03125	0.15	2 < *x*≦3.2
*i* = 6	−0.0078125	0.05	*x* > 3.2
*i* = 7	0	0.0001	*σ*’(*x*)≦0

### Binarization for Filtered Spike Trains

The digital neuromorphic algorithm requires less multiplication operations. Therefore, in this study we use the Otsu’s thresholding method to binarize the filtered spike trains, which can iterate all possible threshold values and compute the expansion measure of each pixel level of the threshold (Otsu, 1978). Therefore, each pixel will fall in either foreground or background. Firstly, separate all the pixels into two clusters based on the threshold as follows

(18) $$\left\{ \begin{array}{c} q_{1}\,\left(t\right)=\sum_{i=1}^{t}p\,\left(i\right)\\ q_{2}\,\left(t\right)=\sum_{i=t+1}^{L}p\,\left(i\right) \end{array}\right.$$

where *p* represents the image histogram. Secondly, the mean of each cluster is calculated by the formulation as follows

(19) $$\left\{ \begin{array}{c} \mu_{1}\,\left(t\right)=\sum_{j=1}^{t}\frac{i\cdot p\,\left(i\right)}{q_{1}\,\left(t\right)}\\ \mu_{2}\,\left(t\right)=\sum_{j=t+1}^{L}\frac{i\cdot p\,\left(i\right)}{q_{2}\,\left(t\right)} \end{array}\right.$$

Thirdly, calculate the individual class variance as follows

(20) $$\left\{ \begin{array}{c} \lambda_{1}^{2}\,\left(t\right)=\sum_{i=1}^{t}{\left[i-\mu_{1}\,\left(t\right)\right]}^{2}\,\frac{p\,\left(i\right)}{q_{1}\,\left(t\right)}\\ \lambda_{2}^{2}\,\left(t\right)=\sum_{i=t+1}^{L}{\left[i-\mu_{2}\,\left(t\right)\right]}^{2}\,\frac{p\,\left(i\right)}{q_{2}\,\left(t\right)} \end{array}\right.$$

Fourthly, square the difference between the means formulated as follows

(21) $$\begin{array}{c} \lambda_{b}^{2}\,\left(t\right)\,\_=\lambda^{2}-\lambda_{w}^{2}\,\left(t\right)\\ \_=q_{1}\,\left(t\right)\,\left[1-q_{1}\,\left(t\right)\right]\,{\left[\mu_{1}\,\left(t\right)-\mu_{2}\,\left(t\right)\right]}^{2} \end{array}$$

where *λ_*b*_*, *λ*, and *λ_*w*_* represent between-class variance, total class variance and within-class variance, respectively. Finally, the formulation can be maximized and the solution is *t* that is maximizing *λ*_*b*_^2^(*t*).

### Considerations for Training With Low Bitwidth Weights

Neuromorphic hardware is largely made out of arithmetic elements and memories. Multipliers are the most space and power hungry arithmetic elements of the digital neuromorphic implementation. The realization of a deep neural network is mainly dependent on matrix multiplications. The key arithmetic operation is the multiply-accumulate operation. The reduction of the precision of the multipliers, especially for the weight matrix, is vital for the efficient realization of deep neural networks. Recent researches have focused on the reduction of model size and computational complexity by using low bitwidth weights of neural networks (Courbariaux et al., 2014). Other neuromorphic hardware systems implement bistable synapses based on a 1-bit weight resolution, which is shown to be sufficient for memory formation (Bill, 2010). However, the models do not only use spike timings, but also use additional hardware resources to read the postsynaptic membrane potential (Sjöström et al., 2001). Therefore, this study trains the proposed DEP algorithm using dynamic fixed point representation. In dynamic fixed point, each number is represented as follows

(22) $${\left(-1\right)}^{s}\cdot 2^{-F\,L}\,\sum_{i=0}^{B-2}2^{i}\cdot x_{i}$$

where *B* represents the bit-width, *s* the sign bit, *FL* is the fractional length, and *x* the mantissa bits.

**FIGURE 5:**
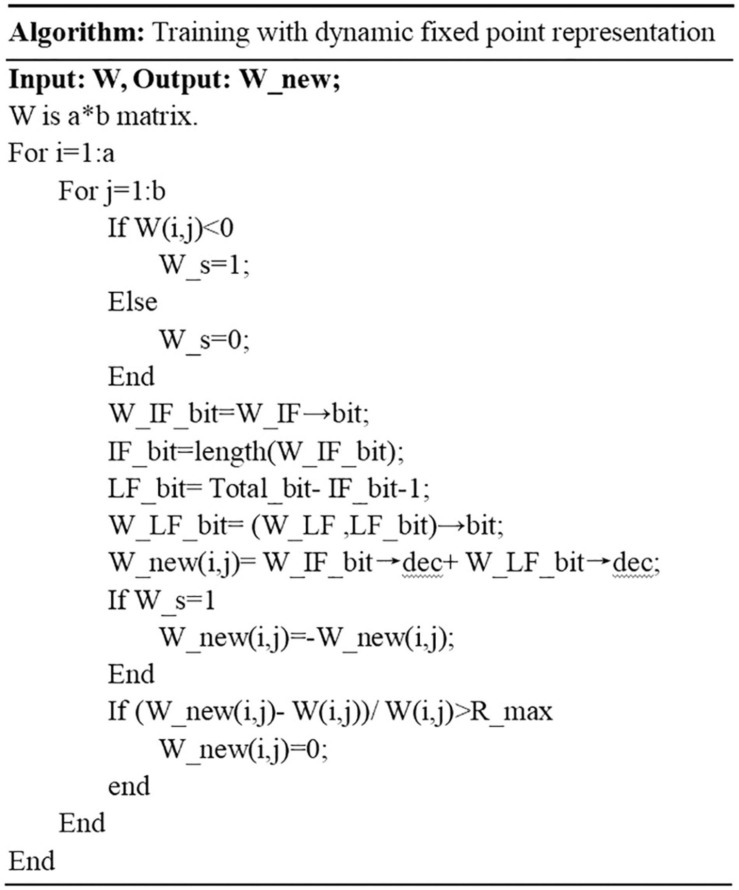
Pseudocode of the algorithm for training with dynamic fixed point representation.

The proposed algorithm is presented in Figure 5. In the pseudo code, the synaptic weight matrix W is the input of the algorithm. Total_bit represents the total bit width of the fixed-point number, and IF_bit is the integer bitwidth. The fractional bitwidth is represented by LF_bit. The integer and fractional parts are represented by W_IF and W_LF. The binary integer and fractional parts are represented by W_IF_bit and W_LF_bit, respectively. The symbol bit is represented by W_s, and R_max defines the fault-tolerant ratio. The error rate refers to the difference between the binary number and the original decimal number divided by the original decimal number. If the error rate exceeds the defined fault-tolerant rate, a specialized process will be used for the binary number. Since the large error occurs in the situation when the considered number is close to 0, this number will be set to 0 if the error rate exceeds R_max. The term *W* is an *a*^∗^*b* synaptic weight matrix to be trained. The first loop is in the line 2. This loop is in the line 2, which is the row loop of the matrix. The second loop is in the line 3, which is the column loop of the matrix. There are two judgments in the proposed algorithm. The first judgment is to determine the symbol bit. If it is negative, then the symbol is 1. If it is positive, then the symbol is 0. The second judgment is to determine the positive and negative when the binary number is converted to decimal number. If the sign bit is 1, it is negative. And it is positive when the sign bit is 0. The third judgment is to consider the error rate between the newly converted number and the original number. If the error rate exceeds the fault-tolerant ratio, the newly converted number will be replaced by 0 for efficient calculation on neuromorphic systems. Finally the updated synaptic weight matrix W_new is output by the processing of the proposed algorithm. By using the proposed algorithm, the memory usage on hardware can be optimized and the energy efficiency of neuromorphic systems can be further improved.

## Results

**FIGURE 6:**
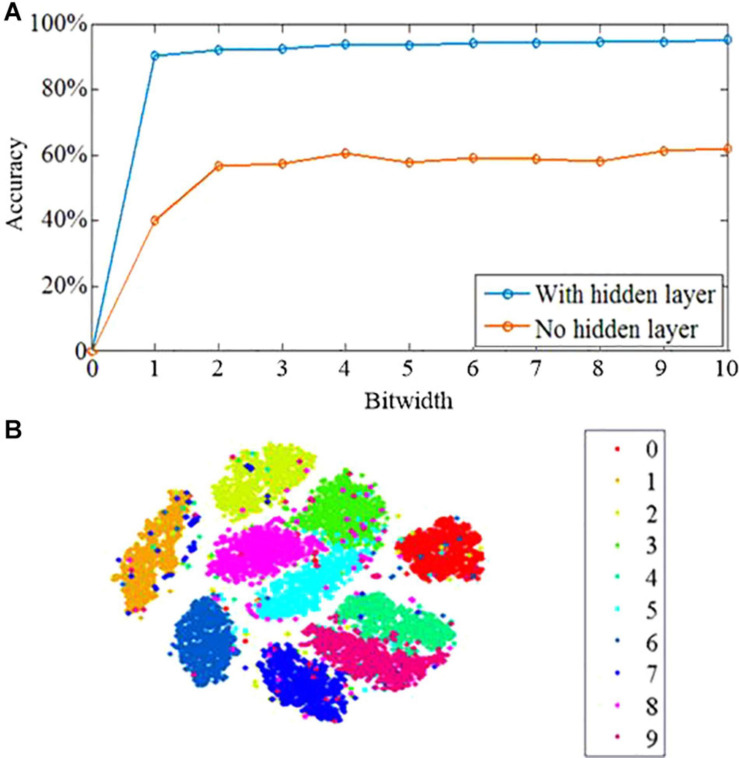
Learning performance of the DEP algorithm. **(A)** The learning accuracy across 10 epochs of training. **(B)** Results of t-SNE dimensionality reduction applied to the activity patterns of the hidden layer after 10 epochs of training.

To demonstrate the effectiveness of the proposed learning algorithm, the standard Modified National Institute of Standards and Technology database (MNIST) benchmark is employed. The MNIST dataset contains 70,000 28 × 28 images of handwritten digits. The image number in the training and testing sets are 60,000 and 10,000, respectively. The dataset is divided into 10 categories for 10 integers 0–9, and each image has an associated label. We trained the networks with no hidden layer, with one hidden layer and two hidden layers on the 60,000 MNIST training images for 10 epochs, and tested the classification accuracy using the 10,000 image test set. As shown in Figure 6A, the network with no hidden layer has poor classification performance of 62.1%. In contrast, the three-layer network with hidden layer has an accuracy of 95.1% by the 10th epoch. The proposed network can take advantage of the multi-layer architecture to enhance the learning performance, which is the critical characteristics of deep learning (Bengio and LeCun, 2007). Another critical characteristic of deep learning is the capability to generate representations, which obtains task-related information and ignores irrelevant sensory details (LeCun et al., 2015; Mnih et al., 2015). The t-distributed stochastic neighbor embedding algorithm (t-SNE) is used to investigate the information abstraction of the proposed algorithm. The t-SNE algorithm can reduce the dimensionality of data with the preservation of local structure and nonlinear manifolds in high-dimensional space. It is a powerful approach to visualize the structure of high-dimensional data (Maaten and Hinton, 2008). The t-SNE algorithm is applied to the hidden layer, which shows that the categories are better segregated with only a small amount of splitting or merging of category clusters as shown in Figure 6B. Therefore, the proposed algorithm has the capability of learning the developing representations in the hidden layer, in which the categories are quite distinct. It reveals that the proposed algorithm can be applied in a deep learning framework. In addition, the proposed algorithm relies on the phenomenon of feedback alignment, in which the feed-forward system comes to align with the feedback weights so that a useful error signal is provided.

**FIGURE 7:**
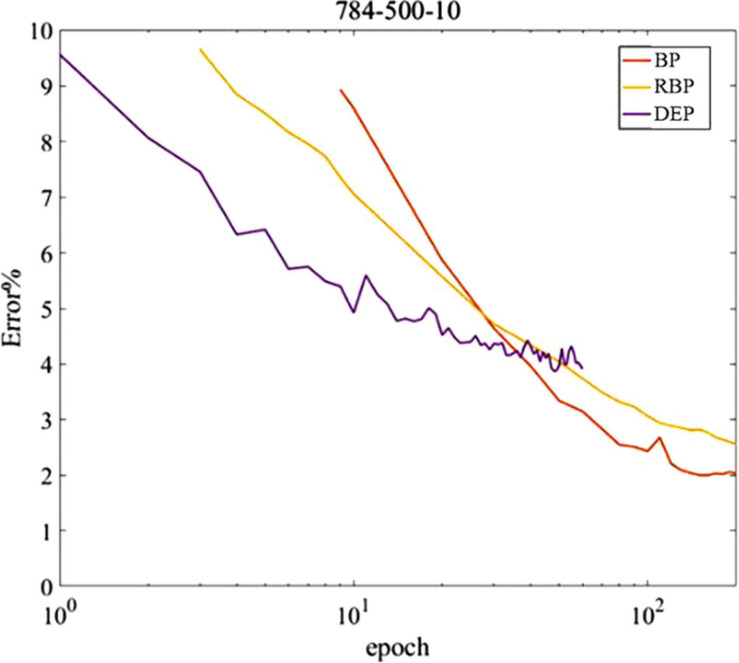
MNIST classification error based on spiking neural network with DEP learning rule and fully connected artificial neural networks with backpropagation (BP) and random backpropagation(RBP) learning rules.

The proposed DEP learning algorithm in a network with one hidden layer trained on permutation invariant MNIST is explored, although it can be generalized to other datasets in theory. Rather than seeking for the optimized classification performance, the equivalent non-spiking neural networks trained by standard BP and random BP are compared with the proposed algorithm, with the parameters tuned to obtain the highest classification accuracy in the current classification task. Weight updates are conducted during each digit input into the spiking network, which is different from the batch gradient descent that performs weight updates once per the entire dataset. As shown in Figure 7, the DEP algorithm requires fewer iterations of the dataset to obtain the peak classification performance in comparison with the two alternative methods. The reason is that the spiking neural network with DEP algorithm can be updated multiple times during each input, which results in faster convergence of learning. In addition, for the equivalent computational resources, online learning with gradient descent strategy has the capability to deal with more data samples and requires less on-chip memory for implementation (Bottou and Cun, 2004). Therefore, for the same number of calculation operations per unit time, online gradient-descent-based learning converges faster than batch learning. Since potential applications of neuromorphic hardware is with real-time streaming data, it is essential for the online learning with DEP algorithm.

**FIGURE 8:**
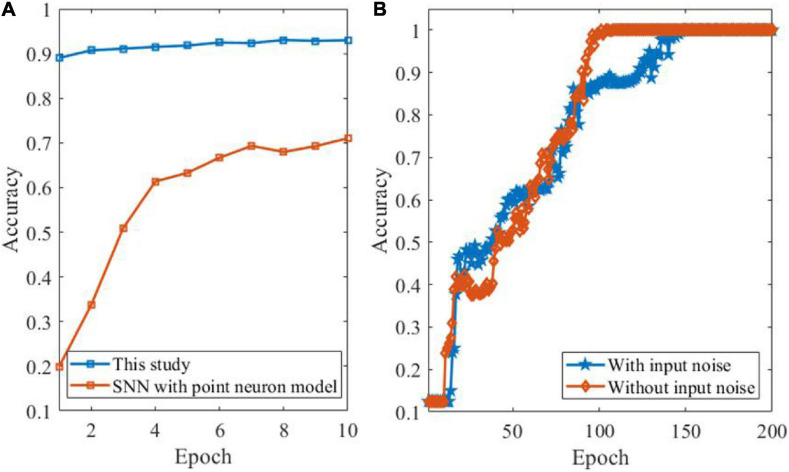
Learning performance by comparison and based on feature detection task. **(A)** Comparison between the proposed DEP algorithm with two layers and SNN with point LIF neuron model. **(B)** Performance evaluation of feature detection by DEP algorithm with and without input noise, respectively.

In order to further demonstrate the learning performance of the proposed DEP algorithm, a comparison between the proposed DEP algorithm with two layers and the SNN with point LIF neuron model is presented. As shown in Figure 8A, the learning accuracy of the SNN model with dendrites, i.e., the proposed DEP algorithm, is higher than the conventional SNN with point neuron model. Besides, we further apply the DEP algorithm in the feature detection tasks to see whether the proposed algorithm could also learn feature detection maps from continuous sensory streams. Previous study has shown that SNN models can defect features from background activities (Masquelier et al., 2008). In order to provide a good benchmark for the proposed DEP algorithm on the feature detection task, the ability of the DEP algorithm is examined for feature detection tasks. In this task, there are eight categories, and each category represents on direction, including 0°, 22.5°, 45°, 67.5°, 90°, 112.5°, 135°, and 157.5°. Each image consists of 729 (27 × 27) pixels. Besides, 10% pixels are randomly selected to add Gaussian noise to make the data set with input noise. Figure 8B shows the learning performance of the DEP algorithm. It reveals that the DEP algorithm can successfully detect feature patterns contaminated by background noise using spike-based framework.

**FIGURE 9:**
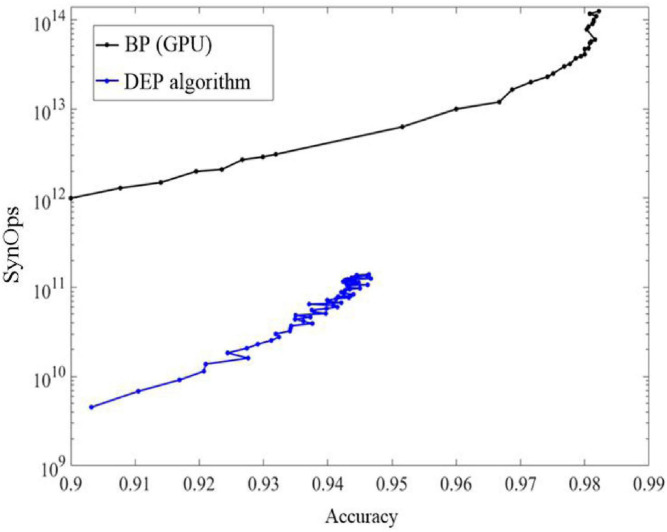
The comparisons of accuracy and SynOps between the proposed DEP algorithm and BP algorithm on graphical processing unit (GPU).

As shown in Figure 9, learning on neuromorphic system can be energy efficient by using the proposed DEP algorithm, because only active connections in the network induce synaptic operations (SynOps) operation. In order to show the learning efficiency, the number of multiply-accumulate (MAC) operations using the BP algorithm is compared with SynOps number with the proposed algorithm. This advantage is critical and promising for neuromorphic computing because SynOps in a dedicated neuromorphic system use much less power than MAC operations on a GPU platform. The learning accuracy of the proposed algorithm increases quickly but the final accuracy is lower than an ANN.

**FIGURE 10:**
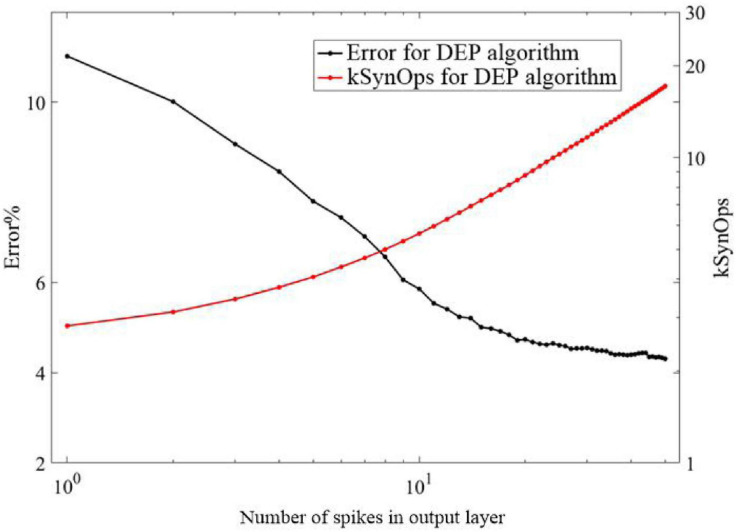
Classification error in the proposed DEP network as a function of the number of spikes in the output layer, and total number of synaptic operations incurred up to each output spike.

As shown in Figure 10, the response of the proposed DEP algorithm after stimulus onset is one synaptic time constant. It leads to 11% error and improves as the spikes number of the neurons in the output layer increases. Classification using the first spike induced less than 20 k SynOps events, most of which exist between the input and hidden layer. In the state-of-the-art neuromorphic system, the energy consumption of a synaptic operation is around 20 pJ (Merolla et al., 2014; Qiao et al., 2015). On such neuromorphic system, single spike classification based on the proposed network can potentially induce 400 pJ, which is superior to the state-of-the-art work in digital neuromorphic hardware (≈2 μJ) at this accuracy (Esser et al., 2016) and potentially 50,000 more efficient than current GPU technology. In addition, an estimation of the power consumption during training by using BP and the proposed DEP algorithm is also presented according to Figure 9. It reveals that about 10^11^ SynOps is cost by the end of 60 epochs, therefore about 33 mJ is cost in an epoch during training. Previous study has demonstrated that 1,000–5,000 mJ will be cost by BP algorithm on conventional GPU platform (Rodrigues et al., 2018). Therefore, there is a 96.7–99.3% reduction for the power consumption by the proposed DEP algorithm during training. The reasons for the low energy cost can be divided into three aspects. Firstly, the segregated dendrite can generate a plateau potential within 50–60 ms, which determines the training time of the proposed network. The training time can be thereby reduced in this way, which can cut down the number of spikes with the decreasing of the training time for each image. Secondly, the conventional BP algorithm induces a trend to make neurons spike with maximum firing rate, and induces synchronization within layers. This means a larger number of spikes. Thirdly, the communication between layers in the proposed algorithm uses a Poisson filter, and *Φ*_*max*_ is set to be 0.2. These results suggest that the proposed DEP learning algorithm can take full use of the spiking dynamics, with the learning accuracy comparable to the spiking network that is trained specifically for single spike recognition in previous study (Mostafa, 2017).

**FIGURE 11:**
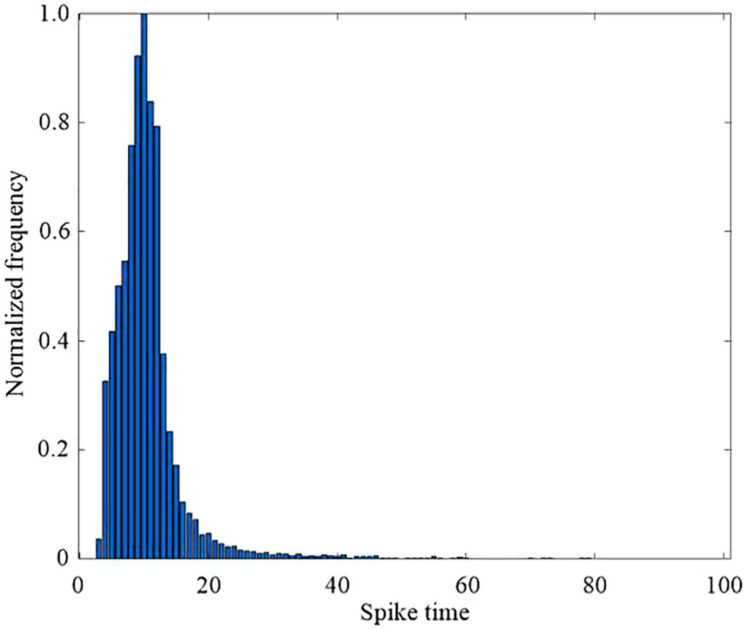
Histograms of spike times in the output layer spike across the 10,000 test set images.

Figure 11 shows the distribution of spike times in the output layer, which is the times at which the proposed SNN makes a decision for all the 10,000 test set images. The proposed SNN with DEP algorithm makes a decision after most of the hidden layer neurons have spiked. The network is thus able to make more accurate and robust decisions about the input images, based on the plateau potentials generated by the dendrites in the proposed DEP algorithm.

**FIGURE 12:**
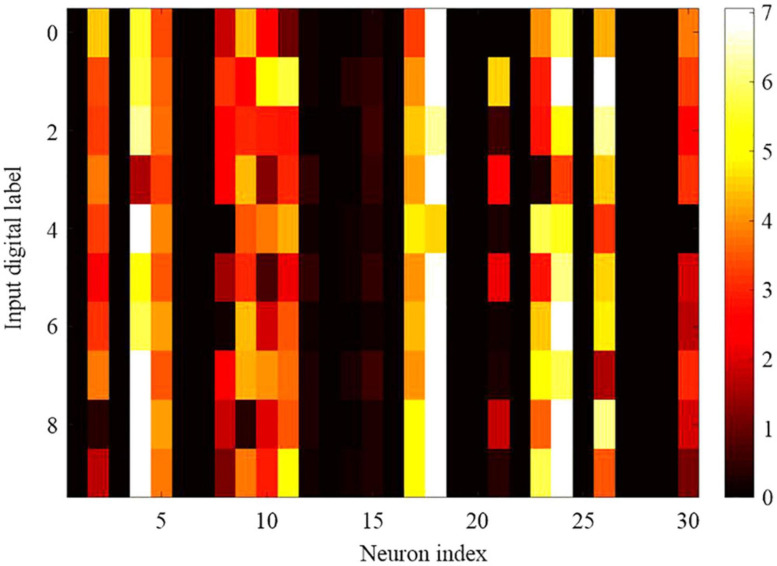
Selectivity and tuning properties of 30 randomly selected hidden neurons in the proposed SNN network with DEP algorithm. It is plotted by heat map with color called YlOrRd, whose color gradually changes across yellow, orange, and red.

As shown in Figure 12, **30** neurons in the hidden layer are selected randomly to explore the selectivity for 10 categories of MNIST data set. The negative log probability for each of the 30 neurons to spike for each of the 10 categories is explored, which means the negative log probability for a neuron to participate in the classification of a specified category. Probability is calculated from the response of the SNN to the 10,000 test digits. It reveals that some neurons are highly selective, while most of the neurons are more broadly tuned. Some of the neurons are mostly silent, but all the neurons in the SNN model contribute to at least one category of classification with the 10,000 test digits. In other word, neurons are typically broadly tuned and contribute to the classification of more than one categories.

**FIGURE 13:**
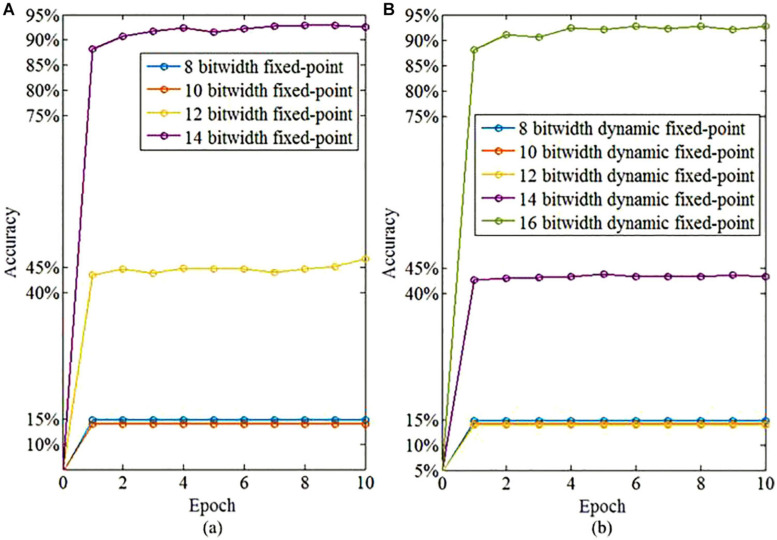
The learning accuracy based on fixed-point and dynamic fixed-point representations. **(A)** The learning accuracy based on fixed-point representation of fractional part with different bitwidth. **(B)** The learning accuracy based on dynamic fixed-point representation with different bitwidth.

We further investigate the necessary bit widths of the fixed-point and dynamic fixed-point, respectively. The bit width of the integer part using the fixed-point calculation is set to 8 to avoid the overflow problem during computation. In contrast, the dynamic fixed-point is not required to determine the bit width of either integer or fractional part. As shown in Figure 13, the fixed-point representation of the fractional part requires 14 bits to obtain a satisfied learning performance that exceeds 90%. Therefore, the satisfied total bit width for fixed-point representation is 22 bit (8 bit for integer part and 14 bit for fractional part). The dynamic fixed-point representation just needs 16 bits to realize high-performance learning. Therefore, the dynamic fixed-point representation in the proposed algorithm provides an efficient approach to reduce the computational hardware resource cost and power consumption for neuromorphic computing.

**FIGURE 14:**
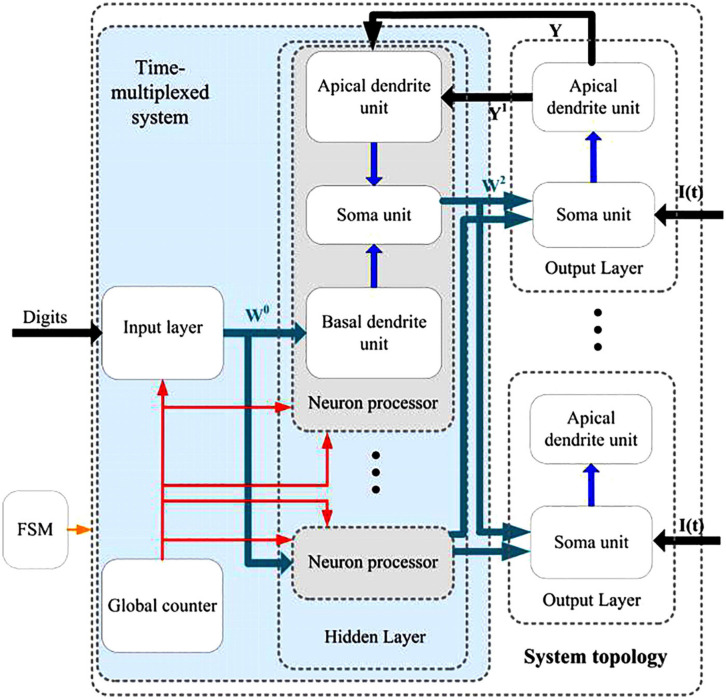
Top-level entity for the neuromorphic architecture of the proposed learning algorithm.

Figure 14 shows the digital neuromorphic architecture at the top level, which contains an input layer, a hidden layer with five physical neural processors, and an output layer with 10 physical neural processors. The input layer and hidden layer are all implemented to use time-multiplexing. The global counter processors the time-multiplexed input neurons and hidden layer neurons sequentially. The FSM module represents the finite-state machine which controls the timing procedure of the whole neuromorphic system. Three parts are contained in the neuron processor in the hidden layer, which are apical dendrite unit, soma unit and basal dendrite unit. The neuron processor in the output layer consists of two parts that are apical dendrite unit and soma unit. The input of the teaching current *I*(*t*) is also mastered by the FSM. The green arrows represent the synaptic connections with learning mechanisms, and black arrows describe the invariant synaptic coupling.

**FIGURE 15:**
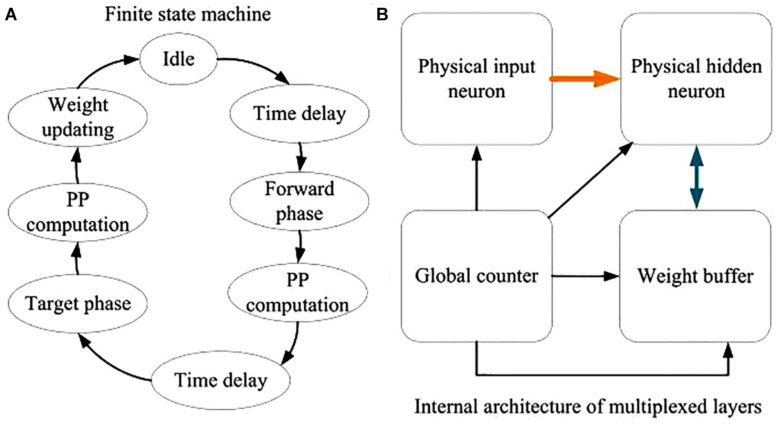
Descriptions of the digital neuromorphic architecture. **(A)** The FSM diagram. **(B)** The internal architecture of the time-multiplexed system.

The detailed description of the FSM is shown in Figure 15A. There are eight states in the FSM diagram, including idle, first time delay, forward phase, first plateau potential (PP) computation, second time delay, target phase, second PP computation and weight updating. By using the FSM controller, the digital neuromorphic system can operate in high performance with definite timing sequence. Figure 15B depicts the internal architecture of the time-multiplexed system. It consists of a physical input neuron, two physical hidden neurons, a global counter, and two weight buffers for each physical hidden neuron. The global counter processes the time-multiplexed physical input and hidden neurons sequentially. The weight buffers store the synaptic weights of the physical neurons. The input digit signals remains available until all the time-multiplexed physical neurons finish their computation. We can also employ the pipeline architecture, by which the maximum operating frequency of the neuromorphic system can be further enhanced.

## Discussion

This study presents a multi-layer feed-forward network architecture using segregated dendrites and the corresponding two-phase learning scheme. Specifically, a piecewise linear approximation and a dynamic fixed-point representation are first introduced in the dendritic learning framework for cost and energy efficient neuromorphic computing. It relies on the feedback alignment phenomenon, in which the feed-forward weights are aligned with the feedback weights to provide useful error signals for learning. The model is optimized for the efficient neuromorphic realization by using the PWL approximation, as well as the binarization for synaptic events. A dynamic fixed-point representation technique is further presented to optimize the proposed DEP algorithm. It reveals that the proposed algorithm with hidden layers can induce higher learning performance, which means that it contains the deep learning capability. In addition, the energy efficient property is proven by comparison with the conventional artificial neural network with BP algorithm. The reasons for this result are likely due to two reasons. First, the gradient descent algorithm suffers from the local optimization problem. When the local optimization is realized, the global optimization cannot be obtained. Spikes emanating from error-coding neurons will be so sparse toward the end of the training that it will prevent the successful adjustments of the weight. The low learning rate will aggravate this problem. A scheduled adjustment of the error neuron sensitivity may solve this problem. Second, the proposed algorithm has not fully utilized the nonlinear dynamics of the neural dendrite. The dynamics of the dendritic compartment have the capability of predicting the dynamics, which may help to improve the performance when considering the dendritic prediction feature. These two methods of modifications, as well as more complicated learning rules, such as momentum or learning rate decay, are left for future work. The output layer neurons spike after the spiking activities of the most neurons in the hidden layer, thus induce a more accurate and robust classification results. The broadly tuned property of the neurons in the hidden layer of the proposed SNN shows that the proposed DEP algorithm can engage each neuron to participate in the classification task. In addition, it shows the superior performance by using the proposed dynamic fixed-point representation by comparing it with the traditional fixed-point computation, which shows that the proposed method can reduce the hardware resource cost considerably. Therefore, our study demonstrates a biologically plausible learning algorithm in a neuromorphic architecture, and realizes the efficient learning by using the DEP approach. In summarize, the proposed DEP algorithm has four aspects of advantages. Firstly, the proposed DEP algorithm cost less SynOps number in comparison with the conventional BP algorithm as shown in Figure 8. It means less power consumption can be realized on neuromorphic hardware. Secondly, faster learning speed can be achieved by the DEP algorithm shown in Figure 7, which is meaningful for on-chip online learning. Thirdly, the solution of credit assignment by dendrites is a vital mechanism for learning in human brain. Therefore, the proposed DEP algorithm is more biologically plausible, which is also a significant ambition of neuromorphic computing. Fourthly, the proposed DEP algorithm is more useful for the online learning with network architecture using more than one layer. As shown in Figure, single point neuron model is not suitable for learning with gradient descent when the network layer number increasing to two.

In the field of neuromorphic computing, neuromorphic systems with on-line learning ability provide a platform to develop brain-inspired learning algorithms, which strive to emulate in digital or analog technologies human brain properties. Online learning requires to be realized based on the input of asynchronous and event-based sequential data flow. Since neuromorphic computing supports continual and lifelong learning naturally, this study presents a SNN model that can deal with the asynchronous event-based spatio-temporal information, which is applicable for neuromorphic systems directly. It provides a novel view for neuromorphic online learning and continual learning, which is meaningful to bridge the gap between neuroscience and machine intelligence. Previous studies have presented a number of neuromorphic systems equipped with synaptic plasticity for general-purpose sensorimotor processors and reinforcement learning (Neftci, 2013; Qiao, 2015; Davies et al., 2018). However, current neuromorphic computing ignores the learning capability to further improve the deep learning performance. Inspired by other neuromorphic studies, more low-power and high-speed techniques can be considered in the future work to obtain a better learning effect.

Previous studies have proposed new algorithms, including attention-gated reinforcement learning (AGREL) and attention-gated memory tagging (AuGMEnT) learning rules, explaining the mechanism of the reinforcement learning optimization in a biologically realistic manner using synapses in deep networks (Roelfsema and Ooyen, 2005; Rombouts et al., 2015). The feedback coupling strength is proportional to the feed-forward strength in these models, which means the learning principles are computationally equivalent to the error back-propagation. It indicates the human brain can solve the credit-assignment problem in a manner that is equivalent to deep learning. However, AGREL algorithm uses the top-down probabilistic model to compute rather than the description and representation of learning from the neural dynamics point of view. There is also no bottom-to-top modeling using spiking neurons in AuGMEnT algorithm. Thus, these two algorithms cannot be employed in neuromorphic computing. Interestingly, we can combine these two algorithms with the presented DEP algorithm to improve the learning performance further.

Efficient learning to solve the credit assignment problem is helpful for the performance improvement of deep learning. This study presents the DEP algorithm for neuromorphic learning, which is meaningful for the communities of both neuromorphic engineering and deep learning. Recently, neuromorphic computing has wide applications. Neuromorphic vision sensors capture the features of biological retina, which has changed the landscape of computer vision in both industry and academia (Chen et al., 2019; Zhou et al., 2019). Although neuromorphic systems with deep learning capability are still in research phases, the development of neuromorphic computing is calling for more biologically realistic processing strategies. Looking forward, with such systems with learning ability, the bridges between machine and biological learning can translate into adaptive and powerful embedded computing systems for a wide category of applications, such as object recognition, neuro-robotic control, and machine learning.

## Conclusion

This paper presented a biologically meaningful DEP algorithm with dynamic fixed-point representation, as well as its digital neuromorphic architecture on LaCSNN. The PWL approximation method and the binarization approach for synaptic events are used in the proposed algorithm for the optimization of efficient implementation. Experimental results show that the learning performance of the proposed DEP algorithm can be improved by adding a hidden layer, which shows the deep learning capability of DEP. Different levels of dendrite segregation will influence the learning accuracy of the network, and the manners of the synaptic feedback connections also play vital roles in the learning performance. By using the fixed-point representation in this work, the hardware resource cost can be cut down by reducing the bit width of the computational elements. This study provides a bridge between the biological learning and neuromorphic learning, which can be used in the applications including object recognition, neuro-robotic control, and machine learning.

## Data Availability Statement

Publicly available datasets were analyzed in this study. This datacan be found here: MNIST http://yann.lecun.com/exdb/mnist/.

## Author Contributions

SY, TG, and BL-B developed the theoretical approach for DEP algorithm with spiking neurons. TG and ZH implemented the source code. JW and BL revised the manuscript and made critical suggestions on this work. SY wrote the manuscript. All authors contributed to the article and approved the submitted version.

## Conflict of Interest

The authors declare that the research was conducted in the absence of any commercial or financial relationships that could be construed as a potential conflict of interest.
